# Orthotopic Transplantation of Human Paediatric High-Grade Glioma in Zebrafish Larvae

**DOI:** 10.3390/brainsci12050625

**Published:** 2022-05-10

**Authors:** Susanna Larsson, Petronella Kettunen, Helena Carén

**Affiliations:** 1 Sahlgrenska Center for Cancer Research, Department of Laboratory Medicine, Institute of Biomedicine, Sahlgrenska Academy, University of Gothenburg, 405 30 Gothenburg, Sweden; susanna.m.larsson@gmail.com; 2 Department of Psychiatry and Neurochemistry, Institute of Neuroscience and Physiology, Sahlgrenska Academy, University of Gothenburg, 413 45 Gothenburg, Sweden; petronella.kettunen@neuro.gu.se; 3 Department of Neuropathology, Nuffield Department of Clinical Neurosciences, University of Oxford, John Radcliffe Hospital, Oxford OX3 9DU, UK

**Keywords:** paediatric glioblastoma, paediatric high-grade glioma, glioma stem cells, zebrafish larvae, in vivo model, experimental model, cancer stem cells, epigenetics

## Abstract

Brain tumours are the most common cause of death among children with solid tumours, and high-grade gliomas (HGG) are among the most devastating forms with very poor outcomes. In the search for more effective treatments for paediatric HGG, there is a need for better experimental models. To date, there are no xenograft zebrafish models developed for human paediatric HGG; existing models rely on adult cells. The use of paediatric models is of great importance since it is well known that the genetic and epigenetic mechanisms behind adult and paediatric disease differ greatly. In this study, we present a clinically relevant in vivo model based on paediatric primary glioma stem cell (GSC) cultures, which after orthotopic injection into the zebrafish larvae, can be monitored using confocal imaging over time. We show that cells invade the brain tissue and can be followed up to 8 days post-injection while they establish in the fore/mid brain. This model offers an in vivo system where tumour invasion can be monitored and drug treatments quickly be evaluated. The possibility to monitor patient-specific cells has the potential to contribute to a better understanding of cellular behaviour and personalised treatments in the future.

## 1. Introduction

Among solid childhood tumours, brain tumours, including high-grade gliomas (HGG), are the leading cause of death. Although HGGs are rare and account for only 3% of all brain tumours in children, the disease is aggressive and incurable, with a 5-year survival of only 5% [1]. The low incidence and ethical issues concerning long-term side effects and difficulties around children limit the number of clinical trials that can be performed. Therefore, better pre-clinical models to identify and evaluate new treatment approaches are needed.

HGGs with fast disease progression and short survival time demand relevant, fast and reliable xenograft models that can provide patient-specific information, as well as information about tumour biology, molecular behaviour and/or treatment responses. Another reason for novel models to study treatment response is the importance of preventing unnecessary side effects from treatment that will cause injury to the developing brain of a child. More relevant primary cell cultures and animal models for paediatric gliomas are of great importance, since it is well known that the genetic and epigenetic mechanisms behind adult and paediatric disease differ greatly [2,3,4].

Studying HGG with its invasive nature in its normal context within the microenvironment and supportive tissue is important for improving treatment strategies. To date, immunodeficient mice are commonly used for xenotransplantation of glioma cells to study the biological behaviour of the tumours and the response of drug treatments [5,6,7,8]. In the case of glioma, however, an in vivo model with shorter time course would be more optimal and beneficial since existing models demand long follow-up times since the progression of the tumour can take up to a year [9,10]. In addition, mouse models are more technically challenging and require expensive housing in advanced facilities. 

An alternative to the commonly used mouse model is the zebrafish (*Danio rerio*) that has been implemented in glioma research over the past decade, although these models have been developed and optimised for adult glioma. The strengths of the zebrafish are many; for example, of the disease-related genes in humans, 82% have been found to have at least one ortholog in zebrafish [11]. Zebrafish embryos and larvae are transparent, which allows for cell tracking over time, and fish can be kept in a dish, making them easy to handle and monitor. Moreover, drugs can be delivered directly to the water where the embryos are kept. They also lack a developed immune system at the early larval stage, which enables xenotransplantation without immunosuppression in young animals [12]. The zebrafish has become an important animal used in mutant and transgenic tumour models [13,14,15]. These models are useful to understand cancer biology, and the development of xenograft models using human tumour cells offer further clinical relevance. However, to our knowledge, no patient-derived xenotransplantation (PDX) models for paediatric gliomas have been developed in the zebrafish so far. Studying human brain cells xenotransplanted into zebrafish larvae has proven to be challenging. Firstly, the optimal holding temperatures for human cells and zebrafish differ, as human cells grow best at 37 °C and zebrafish are most commonly held at 28.5 °C. However, zebrafish larvae can survive and develop in temperatures up to 34 °C [16,17,18,19], and most experiments have been performed with an elevated temperature of around 32–36 °C [20,21]. However, the growth and development of transplanted cells at these temperatures have not been comprehensively investigated and compared. 

Since access to appropriate primary glioma cells can be challenging, many experiments have made use of commercially available glioma cell lines. For example, Hamilton et al. described the growth pattern of two well-known glioma cell lines (U87 and U521) and the microglia response into the invasion point at 4 days post-injection (dpi) and 9 dpi [22,23]. In a study by Vittori et al., the glioblastoma (GBM) cell lines U87 and U343 were assessed upon their interactions with mesenchymal stem cells after transplantation into the zebrafish larvae brain [24]. Although these cell lines could provide important knowledge regarding glioma biology, they do not provide the broad genetic and molecular diversity of patient-derived cells, nor could they be used to evaluate patient-specific treatments. Therefore, more recent studies have been focusing on injecting primary tumour cells to better meet clinical needs [25,26]. In a recent publication by Ahlmstedt et al., these issues were addressed, and 11 adult patient-derived cell lines were orthotopically injected into zebrafish larvae. Forming tumours were evaluated based on growth and invasion. Heterogeneity between cell lines was observed, and tumours resembled the mouse PDX counterparts [27]. Such studies emphasise the importance of using primary cell lines in in vivo models to gain better understanding of different drug responses. 

Generating novel drugs is a meticulous and costly process. Therefore, to reduce the time and expenses involved in developing new drugs, repurposing and combining already approved drugs is an attractive alternative. The alkylating agent temozolomide (TMZ) that is currently used for the treatment of glioblastoma [28] has been combined with drugs that alter the epigenetic landscape of the tumour cells. Epigenetic drugs such as DNA (cytosine-5)-methyltransferase 1 inhibitors and histone deacetylase inhibitors in combination with TMZ have been shown to increase treatment response in adult HGG and paediatric medulloblastoma due to synergistic effects of the drugs [29,30,31,32]. To date, evaluation of epigenetic drugs in combination with TMZ has not yet been performed in patient-derived xenograft zebrafish models [14]. 

Here, we show that paediatric patient-derived primary glioma stem cells (GSCs) can be orthotopically injected into the embryonic zebrafish brain for detailed cell analysis, and the model is suitable for analysing drug treatment response. This in vivo model bridges the gap between in vitro screenings and in vivo experiments in vertebrates. This study is, to our knowledge, the first orthotopic xenograft model focusing on paediatric glioma using primary cell lines, making it a clinically relevant tumour model that allows for personalised treatment screening.

## 2. Materials and Methods

**Figure 1:**
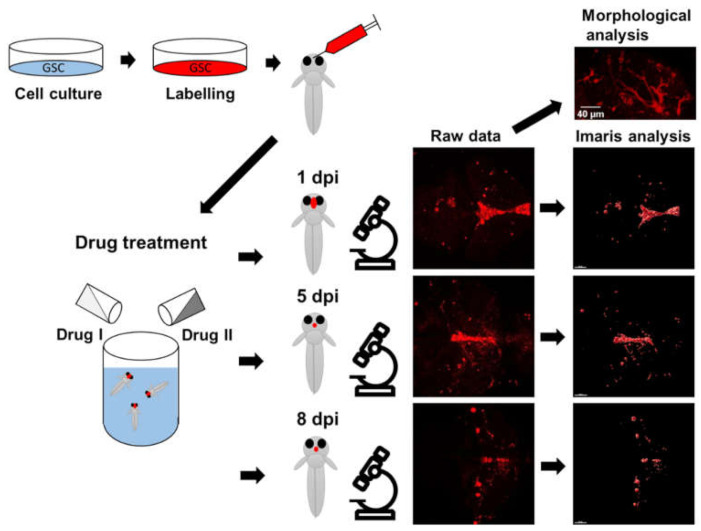
Description of the experimental workflow. Primary paediatric GSCs cultured in 2D were labelled using DiD prior to detachment and injection. Zebrafish larvae were collected at 2 days post-fertilisation and cells were orthotopically injected into the forebrain ventricle, and larvae were kept at 34 °C. Injected GSCs were monitored at 1 day post-injection (dpi), 5 dpi and 8 dpi using confocal imaging when morphology was assessed. Raw data were processed and visualised by the software IMARIS. In vivo treatments were performed by adding drugs to the water of the injected larvae.

The workflow of the experiments is depicted in Figure 1. 

### 2.1. Cell Culture

Primary GSC lines GU-pBT-7, GU-pBT-19, GU-pBT-23, GU-pBT-28 and GU-pBT-58 were cultured as previously described [33]. Cells were either kept at 37 °C or acclimatised by decreasing the temperature by 1 °C per day down to 34 °C. The cells were labelled with CellBrite Cytoplasmic Membrane Dye Red (DiD; Biotium, Fremont, CA, USA) according to the manufacturer’s instructions. Cells were detached using PBS (Hyclone, Logan, UT, USA) and Stempro Accutase (Gibco, Thermo Fisher Scientific Inc., Waltham, MA, USA). Cells were resuspended in Dulbecco’s Modified Eagle Medium/Nutrient Mixture F-12 (DMEM-F12; Gibco) and kept on ice prior to injection. 

GSCs GU-pBT-7 and GU-pBT-19 were transfected using ready-to-use lentivirus/lentiviral particles expressing eGFP under EF1a promoter with puromycin selection marker (Amsbio, Cambridge, MA, USA). Transfection efficiency was 30–45% of all cells. Cells were cultured under selection pressure using medium containing puromycin, which increased GFP expression to ~70%. To confirm proliferation, cells were pulsed with EdU (5-ethynyl-2′-deoxyuridine) 24 h prior to being fixed with 4% paraformaldehyde (PFA) and stained using the EdU Click-iT^®^ EdU Alexa Fluor^®^ 488 Imaging Kit (Invitrogen, Carlsbad, CA, USA).

### 2.2. Animals

Zebrafish (*Danio rerio*) of the AB wild-type line were maintained at the animal facility at the Zoology building at the Department of Biological and Environmental Sciences, University of Gothenburg. Fish were kept at 28.5 °C and at a 14:10 h light:dark cycle, and fed twice per day with GEMMA Micro 300 pellets from Skretting (Stavanger, Norway) and once per day with Artemia nauplii.

### 2.3. Cerebral Injection of Larvae

Fertilised eggs used in embryo/larva experiments were collected in rack system water, and debris was manually cleaned away with a plastic pipette. Eggs were then kept in Petri dishes in embryo media (EM) (5.03 mM NaCl, 0.17 mM KCl, 0.33 mM CaCl_2_•2H_2_O, 0.33 mM MgCl_2_•6H_2_O) at 28.5 °C in an incubator with a 14:10 h light:dark cycle. Larvae were pre-treated with 0.003% *w*/*v* 1-phenyl 2-thiourea (PTU) dissolved in EM and kept in PTU throughout the experiment to prevent pigmentation. GSCs used for orthotopic injections were in passage 15–25. Zebrafish embryos, 2 days post-fertilisation (dpf), were dechorionised if necessary and selected for experiments. Animals were anaesthetised using 0.02% tricaine methanesulfonate (MS 222) dissolved in EM. Larvae were embedded in 1.7% low-melting-point agarose in EM on glass slides.

Cells were loaded into a glass micropipette pulled by a P-97 Flaming/Brown Micropipette Puller (Sutter Instruments Co.) and attached to a Narishige MN-153 micromanipulator (Narshige) and connected to an air-driven Picospritzer microinjector (Parker). Prior to loading cells, the tip of the glass micropipette was carefully broken to avoid cell clumping, and cells were injected at a slow speed. The volumes of injection droplets for each tip and cell line were then measured in mineral oil and the pressure and injection time were adjusted to give the same number of injected cells in all fish. Approximately 100–200 cells were injected at a volume of 1 nl by inserting the needle into the forebrain ventricle. However, we were not able to count the exact number of injected cells right after the injection. For this reason, we used the absolute number of cells at 1 dpi as the most accurate evaluation of cells at baseline. After injection, the animals were removed from the agarose, transferred to fish Ringer’s solution (116 mM NaCl, 2.9 mM KCl, 1.8 mM CaCl_2_, 5 mM HEPES, pH 7.2) containing PTU and placed individually in wells of 48-well plates or in groups of 5 in wells of 24-well plates with a 14:10 h light:dark cycle at 34 °C until imaging. Four animals were removed from the experiment due to death or injury before the second scanning at 5 dpi.

### 2.4. Live Imaging of Larvae

An upright LSM 710 confocal microscope (Carl Zeiss AG, Oberkochen, Germany) was used to acquire images. Animals were anaesthetised using 0.02% MS 222 dissolved in EM before being embedded in 1.7% low-melting-temperature agarose. Images were taken at 1 day post-injection (dpi) and at 5 dpi. DiD-positive cells were visualised at Ex/Em 644/665 nm and GFP-positive cells at Ex/Em 488/510 nm, and a Z-stack of the zebrafish brain was collected at 2 µm by ZEN software (Zeiss).

### 2.5. Imaging Analysis

Imaging analysis software Imaris version 9.7 (Oxford instruments, Abingdon, UK) was used to analyse the image stacks collected from the zebrafish brain imaging. Images were processed in all channels (GFP = green, DiD = red) and volumes as well as number of cells were calculated from the full stack. For counting the number of GSCs, we used the Spots Creation Wizard in Imaris, using a cut-off of 5 µm in diameter to identify individual cells. For volumetric analysis, we used the Surface Creation Wizard giving data on the corresponding volumes. After GSCs were identified with the program, all detected tumour cell volumes were summarised for each fish. The same imaging analysis settings were used for all fish investigated.

Morphological features such as cell shape (round, oblong, drop-shaped and star-shaped), as well as length of cell extensions (no extensions, small, medium length and long extensions) were scored blindly by two researchers from the unprocessed raw stacks. Individual fish could contain more than one morphological feature or extension length, and the presence of these features was scored for each fish.

The number of cells at 1 dpi and 5 dpi, as well as number of cells with either DiD or GFP, were compared with a paired *t*-test. The odds of finding morphological features at either of the time-points were calculated with the MedCalc odds ratio calculator (https://www.medcalc.org/calc/odds_ratio.php (accessed on 28 April 2022)).

### 2.6. Immunocytochemistry

Immunocytochemistry was performed according to the manufacturers’ instructions. Cells were fixed with 4% PFA and stained for the differentiation marker glial fibrillary acidic protein (GFAP) using a monoclonal mouse antibody, Sigma-Aldrich G3893 (1:1000), and incubated overnight. A goat secondary antibody conjugated to Alexa dye 594 (1:1000) (Molecular Probes, Thermo Fisher Scientific Inc., Waltham, MA, USA) was added for 1 h at room temperature and 4′,6-diamidino-2-phenylindole (DAPI) was used as a nuclear counterstain. For imaging and quantification, the Operetta microplate reader (Perkin Elmer, Waltham, CA, USA) was used together with the Harmony software.

### 2.7. Immunohistochemistry

Larvae were freshly frozen after an overdose of 1% MS 222 at 1 dpi, 5 dpi or 9 dpi. For sectioning, a cryostat, Leica CM3050 S, was used to obtain 8 µm sections. Sections were heat-treated using citrate buffer of pH 6.0 and stained with the Vectastain ABC kit (Vector Laboratories, Burlingame, CA, USA) using a human-specific rabbit monoclonal nestin antibody, ab105389 Abcam, (1:300), according to the manufacturer’s instructions.

### 2.8. Drug Treatments

In vitro experiments were performed using adherent cells in 96-well plates. Culture media was changed the day after seeding to media containing TMZ (600 µM, Sigma-Aldrich, St. Louis, MO, USA), valproic acid (VPA) (500–1000 µM, Sigma-Aldrich) or decitabine (2.5–5 µM; Selleck Chemicals, Houston, TX, USA) as single drugs and as combinations of TMZ/VPA and TMZ/decitabine. Decitabine was dissolved in DMSO, and a vehicle DMSO control was therefore included as well as untreated controls. Culture media was changed into new media with the corresponding drugs 48 h after the first treatment, and after another 48 h, cells were fixed in 4% PFA. EdU incorporation assays were performed as described above in Section 2.1.

Prior to the in vivo drug treatments, larvae were tested for temperature and drug tolerance based on survival and malformation using different concentrations of the drugs (data not shown). When the suitable conditions had been established, three animals per treatment group were injected with GSCs and treated with TMZ (100 µM), VPA (100 µM) and decitabine (100 µM) added to the EM. Drug concentrations were decided upon reviewing the literature of the same drugs used in zebrafish previously [22,34,35]. As a control group, DMSO of the highest concentration 0.01% was added to the EM. Drugs were added after injection at 0 dpi and cycled 2 days with drugs + 2 days without drugs + 2 days with drugs. Confocal scans were acquired at 1 dpi, 5 dpi and 8 dpi. 

Two-sample t-tests were used to test for the differences in cell number between control and treatments and combinations. *p*-values of less than 0.05 were considered statistically significant. Using the Bliss independent model [36], synergy between drugs was calculated for the three GSC lines based on cell number ratio.

## 3. Results

### 3.1. Temperature Acclimatisation of GSCs Prior to Injection Causes Morphological Differences and Reduced Survival In Vitro and In Vivo

**Figure 2:**
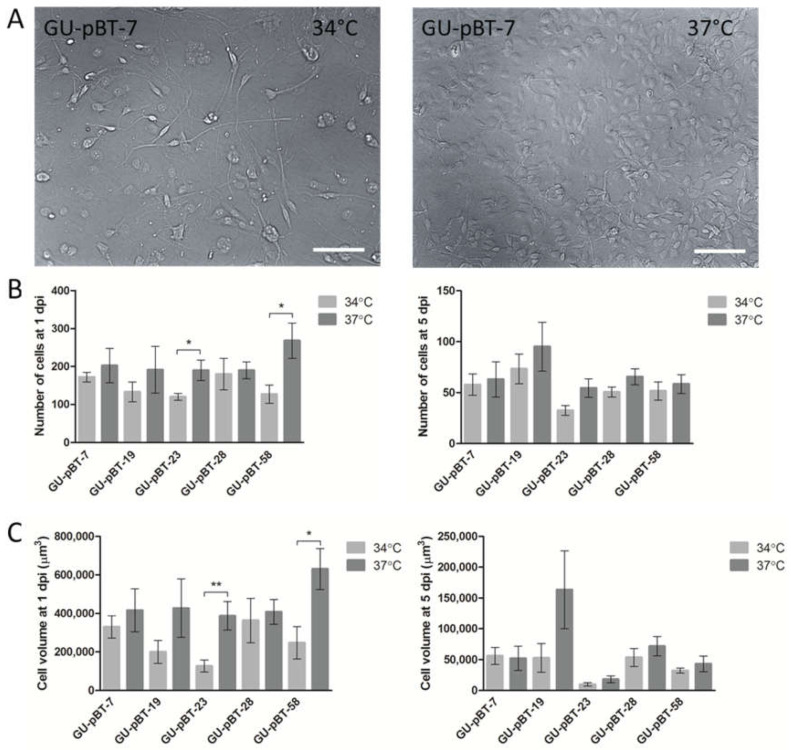
Effect of growing glioma stem cells at different temperatures when studied in vitro prior to transplantation and in vivo post-transplantation into zebrafish larvae. (**A**) Brightfield images of the GSC line GU-pBT-7 grown at different temperatures. Temperature was decreased in vitro from 37 °C to investigate cell growth after injection into the larvae brain (34 °C). Cell growth was decreased by temperature, although cells survived and continued to proliferate. Scale bars = 100 µm. (**B**) Comparison of cell numbers of injected GSC lines into the zebrafish larvae brain was realized to study the importance of acclimatisation of cells in vitro prior to injection. Graphs show the number of tumour cells counted in animals at 1 day post-injection (dpi) and 5 dpi, grown at either 34 °C or 37 °C prior to injection. Each treatment group contained 4–6 fish at 1 dpi and 4–6 fish at 5 dpi. (**C**) Comparison of cell mass volumes of injected GSC lines in zebrafish larvae brains at 1 dpi and 5 dpi. Graphs show volume of cells measured in the same animals as in (**B**). Data are presented as means and standard errors. * *p* < 0.05 and ** *p* < 0.01.

We evaluated cell growth temperatures in culture to determine the optimal conditions for our paediatric GSCs. We first studied the effect of growth and morphology in vitro in the primary paediatric GSC line GU-pBT-7 grown at either 34 °C or standard culture temperature of 37 °C. Cells grown at 34 °C required a longer time to reach confluency compared to cells grown at 37 °C (data not shown). Morphologically, GU-pBT-7 seeded at the same density resulted in fewer cells at passage (day 4–5) and contained more blebbing cells when grown at 34 °C compared to cells grown at 37 °C (Figure 2A).

**Figure 3:**
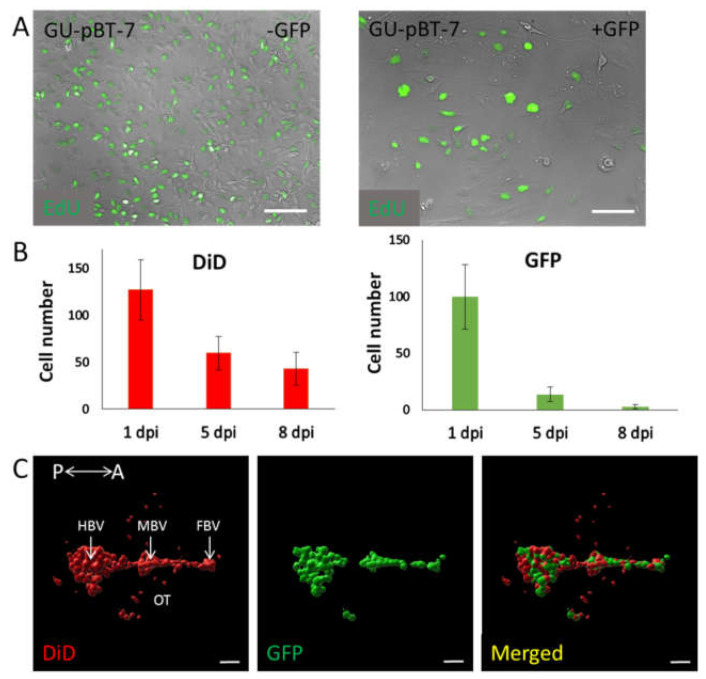
Different labelling methods can be used to visualise cells in the zebrafish brain over time. (**A**) Study of proliferation rate in vitro between unlabelled and GFP-expressing GSC GU-pBT-7 cells. EdU (green) was used to observe cells in S phase. Among unlabelled cells, approximately 80% of all cells were dividing compared to GFP-expressing cells, where proliferation decreased and approximately 30% of all cells were in a dividing state. Scale bars = 100 µm. (**B**) Number of DiD-labelled and GFP-expressing GU-pBT-7 cells in larval brains at 1 dpi, 5 dpi and 8 dpi. GFP-expressing cells were stained with DiD prior to injection. Average data from 28 injected embryos (n(1 dpi): 27 fish, n(5 dpi): 21 fish, n(8 dpi): 8 fish). The comparison between DiD and GFP showed significantly more cells imaged with DiD (1 dpi *p* < 0.001; 5 dpi *p* < 0.001; 8 dpi *p* < 0.01). Graphs show means and standard deviations. (**C**) Representative 3D visualisation using IMARIS software of injected GU-pBT-7 cells in a 3 dpf zebrafish embryo brain at 1 dpi. Panels (left; middle; right) show staining with DiD (red), GFP expression (green) and merged channels, respectively. Scale bars = 50 µm. P: posterior, A: anterior, HBV: hindbrain ventricle, MBV: midbrain ventricle, FBV: forebrain ventricle, OT: optic tectum.

We next injected five primary paediatric GSC lines, GU-pBT-7, GU-pBT-19, GU-pBT-23, GU-pBT-28 and GU-pBT-58, grown at both temperatures into the brain of 2 dpf zebrafish embryos (Figure 2B,C). Since the larvae were kept at 34 °C during the experiment, we investigated possible developmental abnormalities or increased death. We found no effect of the increased temperature on the embryos (data not shown). We did observe a difference in GSC numbers (Figure 2B) and GSC mass volumes (Figure 2C) at 1 dpi for most cell lines, where cells grown at 37 °C prior to transplantation performed better in vivo than those grown at 34 °C. This difference was less prominent at 5 dpi. Moreover, the success rate for injections and larvae survival was high—close to 100% in both conditions (data not shown). Cells were monitored by labelling with DiD prior to detachment and injection (Figure 3).

**Figure 4:**
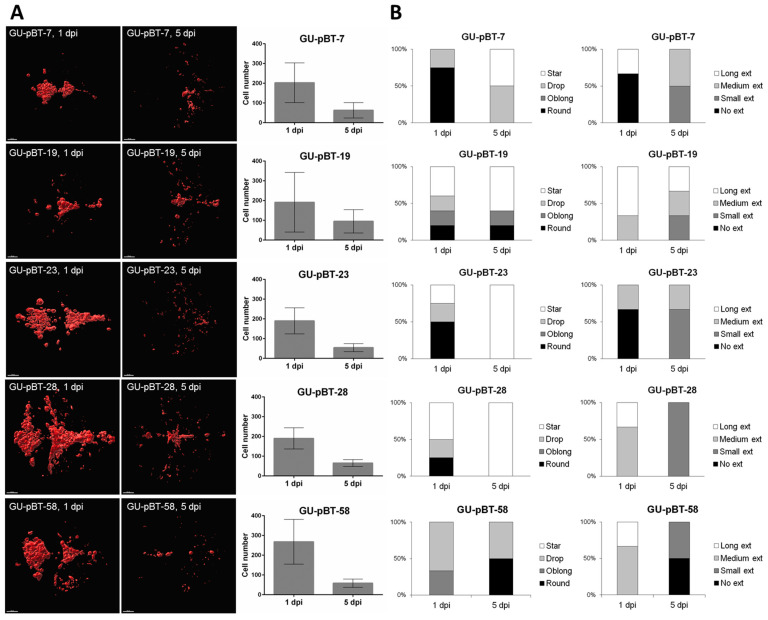
Characterisation of the injected GSC lines. (**A**). Left panels depict maximal injection images of confocal scans of DiD-labelled cells in vivo at 1 day post-injection (dpi) and 5 dpi to illustrate tumour cell burden. Scale bars = 50 µm. Graphs showing number of injected cells at 1 dpi and 5 dpi in vivo as means and standard deviations. Tumour cell mass decreased by more than 50% in all cell lines (GU-pBT-7 *p* < 0.05; GU-pBT-19 *p* = 0.055; GU-pBT-23 *p* < 0.001; GU-pBT-28 *p* < 0.001; GU-pBT-58 *p* < 0.05. (**B**) Morphological description (cell shape and extension lengths) of labelled GSCs at 1 dpi and 5 dpi, presented as the percentage of fish displaying injected cells with the specific morphology. Across all cell lines, cells shifted from more rounded shapes to elongated cell bodies. Cells also tended to develop small extensions over time.

When investigating the morphological differences between injected GSCs from cell culture conditions of 34 °C and 37 °C at 1 dpi, we observed that cells from 37 °C seemed to be more advanced in morphology, resembling differentiated astrocytes with multiple cell structures and longer cell extensions compared to cells grown at 34 °C (Figure 4 and Supplemental Figure S1). Five days after injection, cells more often displayed star-shaped morphology regardless of starting temperature. Most cell lines showed longer cell extensions coming from 37 °C cultures, though the extensions were shorter at 5 dpi compared to at 1 dpi. Based on these results, we chose not to acclimatise GSCs to 34 °C prior to injection.

### 3.2. Evaluation of Labelling Methods of GSC Lines

To guide the following in vivo experiments, we evaluated two labelling methods, i.e., fluorescent protein expression vs. synthetic membrane dye. To this end, we first compared the growth of GU-pBT-7 cells in vitro with/without expression of GFP and visualised the proliferation by EdU staining (Figure 3A). Cell proliferation in culture among unlabelled GSCs was ~80% in contrast to ~30% among GFP-expressing GSCs compared to total number of cells, thereby resulting in a decreased proliferation rate after transfection. Additionally, GFP-expressing cells presented a different morphology in culture with bigger and more blebbing cells. Furthermore, GFP-positive cells were estimated to approximately 70% of the cell culture at harvest upon transfection, while the DiD-labelled cell population was estimated to approximately 95% of total cell count at harvest (data not shown).

Next, GU-pBT-7 cells expressing GFP, labelled with DiD were injected in 2 dpf zebrafish embryos (Figure 3B,C). The result of injected cells showed good coverage of cells at 1 dpi, where most cells injected were positive for both GFP and DiD. However, the total number of DID-labelled cells was significantly higher across the whole experiment (Figure 3B). At 5 dpi, the number of GFP-expressing cells was less than 50% of the DiD-labelled cells still remaining in the larvae brain. Labelling cells using DiD showed a better coverage throughout all time points of the experiment (Figure 3C). We concluded that calculating only GFP-expressing cells, which had a lower labelling efficiency from the start, would possibly result in an inaccurate count of cell numbers and volumes. Based on these results, we chose not to use GFP-expressing GSCs but moved forward with DiD labelling prior to injection.

### 3.3. Evaluating Take-Rate, Cell Survival, Growth and Morphology of a Panel of Primary Paediatric GSC Lines In Vivo

Five primary paediatric GSC lines (GU-pBT-7, GU-pBT-19, GU-pBT-23, GU-pBT-28 and GU-pBT-58) were labelled with DiD and then orthotopically injected into embryonic zebrafish brains (12 fish/cell line) at 2 dpf (Figure 4). No temperature acclimatisation was performed on the cells prior to injection according to data in Figure 2B,C. The success rate for the injections was high, i.e., close to 100%, for all five cell lines at 1 dpi (data not shown). Directly after injection, most of the tumour cells resided in the forebrain and midbrain ventricles (data not shown). At 1 dpi, a portion of the injected cells remained in the ventricles, but some cells were in the process of migrating into the forebrain (telencephalon) and midbrain (mainly optic tectum). Migrating or contact-seeking cells could be found in various places of the brain at both 1 and 5 dpi for all the investigated GSC lines (Figure 4A). Three of the cell lines (GU-pBT-7/19/23) showed a more invasive growth pattern where cells could be observed further from the injection site at 5 dpi. At 5 dpi, the number of cells in the ventricular areas had decreased for all cell lines and cells were also found in the surrounding brain tissue (Figure 4A). The number of GSCs was less prominent at 5 dpi, although all five cell lines were still visible in vivo at this time point. The decrease in cell number between 1 dpi and 5 dpi was more than 50%, though approximately the same number of cells remained in the brain at 5 dpi regardless of the number of GSCs at 1 dpi (Figure 4A).

We studied the injected cells morphologically by grading the cell shapes and cell extension lengths at 1 dpi and 5 dpi (Figure 4B). Cells adjusting to the environment in the larval brain tended to shift from round or oblong cells to more star-shaped morphology. Many of the lines shared similar cell morphology, e.g., there was a mix of different cell types at 1 dpi and an expansion of the star-shaped cells between 1 dpi and 5 dpi. A shortening of extension length could also be observed between 1 dpi and 5 dpi, suggesting a less invasive growth at this stage (Figure 4B). We investigated the odds that fish had star-like cells and long extension at 5 dpi compared to 1 dpi, but these probabilities were not statistically significant.

**Figure 5:**
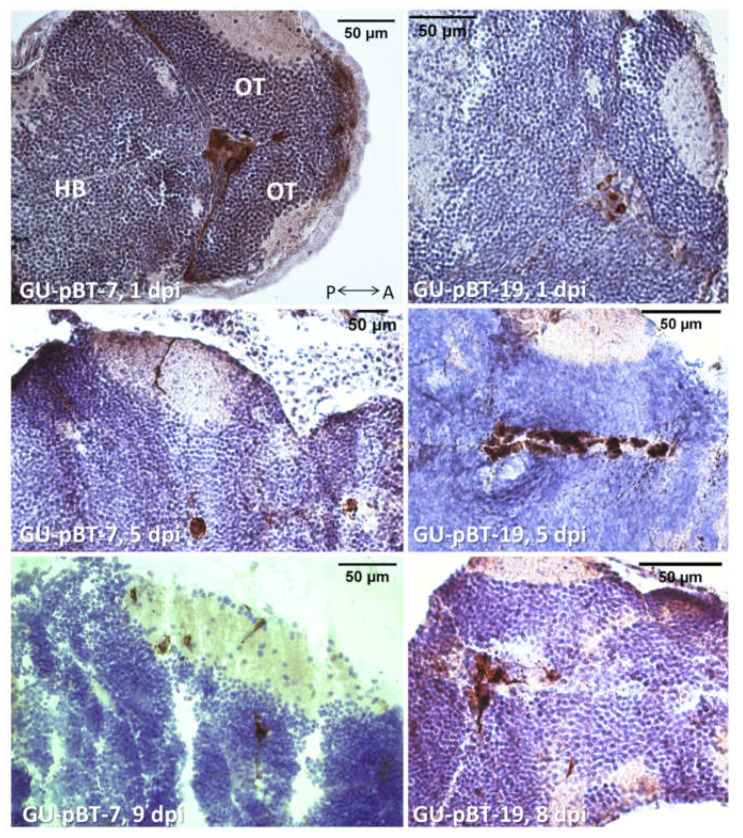
Immunohistochemistry of injected GSCs in the zebrafish brain. Micrographs of human nestin staining (brown) of injected cell lines GU-pBT-7 and GU-pBT-19 at 1 dpi, 5 dpi and 8 dpi. Images show that the human cells remain in the larvae brain up to 8 dpi. A: anterior, P: posterior, OT: optic tectum, HB: hindbrain.

We next confirmed the presence of human tumour cells after injection of GSC lines GU-pBT-19 and GU-pBT-7 in the zebrafish brain using immunohistochemistry (Figure 5). Positive staining of human nestin in GSC-injected fish was detected at 1 dpi, 5 dpi and 9 dpi. At 1 dpi and 5 dpi, injected cells were localised to the forebrain ventricle, where they were delivered initially. At 9 dpi, cells could be observed at the injection site and migrated to other regions of the brain, such as the tectum.

### 3.4. GSCs Respond to Drug Treatments In Vitro

**Figure 6:**
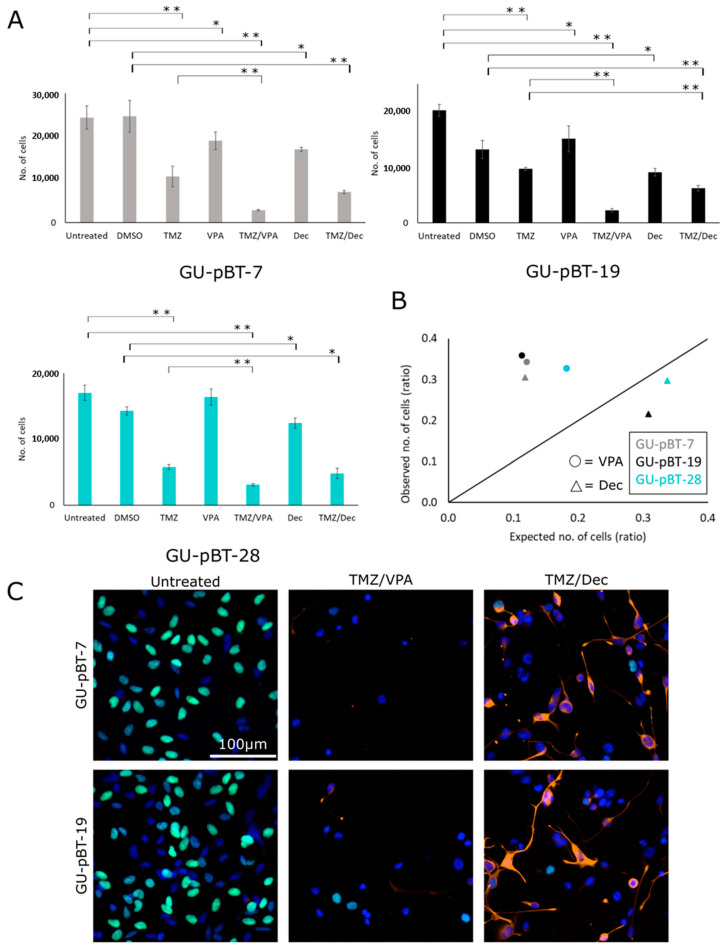
GSC response to drug treatments in vitro with TMZ, VPA and decitabine in vitro, as single treatments and in combinations. (**A**) Number of cells in the different treatments. DMSO: dimethyl sulfoxide; TMZ: temozolomide; VPA: valproic acid; Dec: decitabine. The doses were: GU-pBT-7: DMSO, 0.005%, TMZ 600 μM, VPA 1000 μM and Dec 2.5 μM; GU-pBT-19: DMSO, 0.01%, TMZ 600 μM, VPA 500 μM and Dec 5 μM; GU-pBT-28: DMSO, 0.005%, TMZ 600 μM, VPA 500 μM and Dec 2.5 μM. Data are presented as means and standard deviations. *p*-values between controls and treatments, as well as between TMZ and combinations of TMZ/VPA and TMZ/Dec, are * *p*-value < 0.05 and ** *p*-value < 0.01, respectively. (**B**) Bliss independence model for each GSC line using the combination treatments. Values under the line indicate a positive score (no synergy between the drugs) and values over the line correspond to a negative score (synergy between treatments). (**C**) Immunocytochemical analysis using Harmony software of the GSCs in vitro. Nuclei staining (DAPI; blue) was visible in all treated cells; proliferation (EdU incorporation; green) was mostly visible in untreated cells and differentiation (astrocyte marker GFAP; red) was most pronounced in TMZ/Dec-treated cells. Cell data are quantified in Supplemental Figure S2.

Treatments with either the chemotherapeutic agent TMZ, the histone deacetylase inhibitor valproic acid (VPA) or the DNA methyltransferase inhibitor decitabine were performed on three primary cell lines GU-pBT-7, GU-pBT-19 and GU-pBT-28 cells in vitro (Figure 6). Treatment with TMZ, VPA or decitabine as single agents resulted in a decrease in the number of viable cells for all GSC cultures. Treatment with the TMZ/VPA combination resulted in a significant decrease in cell number compared to treatment with only TMZ. The combination of TMZ/decitabine only showed a significant treatment effect in GU-pBT-19 compared to the TMZ treatment alone, as did some of the combination treatments (Figure 6A). A synergy effect was present between TMZ and VPA in all three cell lines, and the combination of TMZ and decitabine gave a synergistic effect in GU-pBT-7 (Figure 6B).

We further assessed proliferation using EdU incorporation in GU-pBT-7, GU-pBT-19 and GU-pBT-28 following drug treatment. The combination treatment with TMZ/VPA and TMZ/decitabine showed decreased proliferation in both lines (Figure 6C and Supplemental Figure S2). To access the differentiation capacity of decitabine, as a DNA methyltransferase inhibitor of tumour cells, we stained cells treated with TMZ in combination with VPA or decitabine for the astrocyte marker GFAP. Analysis results from the Harmony software confirmed differentiation in GU-pBT-7 and GU-pBT-19 following treatment with decitabine. This differentiation property could not be seen in treatment using VPA (Figure 6C and Supplemental Figure S2).

### 3.5. Xenotransplanted GSCs Respond to Drug Treatments of the Larvae

**Figure 7:**
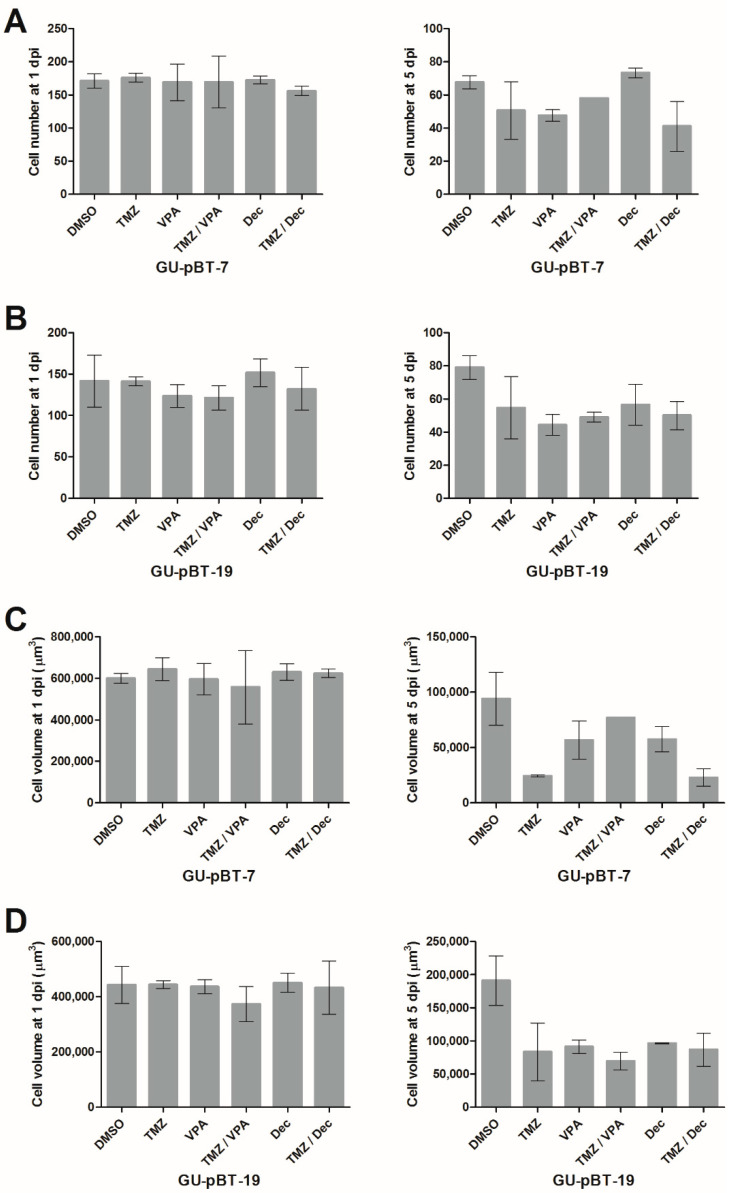
Injected GSCs respond to drug treatment in vivo. Drug response of GSCs injected into embryos based on number (**A**,**B**) and volume (**C**,**D**) of cells detected in the brains at 1 dpi and 5 dpi. Compared to the DMSO control, there was a decrease in cells in the treated GU-pBT-7 and GU-pBT-19-injected fish. Dpi: days post-injection; TMZ: temozolomide; VPA: valproic acid; Dec: decitabine. Data are presented as means and standard errors.

In a pilot experiment, we performed a drug screen using TMZ, VPA and decitabine, previously showing an effect on the GSCs in vitro (Figure 6). Drug treatment was performed by adding dissolved drugs directly into the water of larvae injected with two of the cell lines responding well in vitro, GSC lines GU-pBT-7 and GU-pBT-19. When comparing the GSC number (Figure 7A,B) and cell mass volume (Figure 7C,D) in the brain of drug-treated vs. untreated larvae, we observed no difference at 1 dpi in either GSC line. At 5 dpi, the drugs left a treatment signature at the number of cells, equal for both GU-pBT-7 and GU-pBT-19. Interestingly, drug effects on cell volumes were more prominent than cell numbers, particularly in GU-pBT-19. At 8 dpi, no representative result could be collected since the majority of larvae had died (data not shown). No synergy effects were observed for the in vitro experiment (Figure 6A).

Finally, we explored how the two GSC lines responded morphologically to the different pharmacological treatments in vivo at 1 dpi and 5 dpi (Supplemental Figure S3A,B). At 5 dpi, the epigenetic drugs VPA and decitabine showed signs of having induced differentiation of GU-pBT-7 by increasing the presence of star-shaped cells in the injected fish. GU-pBT-19 cells showed star-like morphology in all drug treatments already at 1 dpi. Similarly, the proportion of GU-pBT-19 cells with long extensions was increased in VPA and decitabine-treated animals at 1 and 5 dpi.

## 4. Discussion

In this study, we developed an orthotopic zebrafish model to study patient-derived paediatric GSCs in vivo up to 8–9 days after injection of cells into the brain. The novelty of this study lies in the use of primary paediatric brain tumours. The lack of accurate models resembling childhood HGGs is a missing piece in the field of brain tumours. We use this novel approach to better represent the diversity and differences between patients. Injected fish were imaged using in vivo confocal microscopy, and cell number, volume and morphology were investigated in detail using state-of-the-art imaging software allowing a 3D analysis of the tumour site. Our experiments showed that take-rate was higher for cells grown at regular temperature (37 °C) rather than at an adjusted, lower temperature (34 °C) prior to xenotransplantation. Moreover, we observed a higher percentage of cells stained using the membrane dye DiD compared to GFP expression following transfection of GSCs. The imaging analysis showed that different GSC lines displayed different morphology in vivo, where cell shape and length of cell extensions varied depending on cell lines. Finally, we explored this model as a patient-specific drug response tool to be used as a guide for treatment plans.

Overall, the findings from the xenotransplanted larvae in this study resonate with our observations of GSCs grown in culture [33] and xenotransplantation into mice [10], where different cell lines promote an individual morphological appearance. The morphological differences between cell lines seen in larvae have previously been observed and evaluated more extensively in mice, where cell-specific growth patterns and migration could be distinguished between the cell lines [10]. The more invasive growth pattern seen in GU-pBT-7/19/23 could also to some extent be seen in the mouse model where GU-pBT-7/23 had the shortest survival time. The cultured GSCs injected into mice also mimicked the originating tumour cells in terms of genetic and epigenetic features, and there was a strong correlation between patient survival and survival of mice [10]. The invasive behaviour of the GSCs used in the present study stands in contrast to our previous experiments using the serum-cultured cell line U87 where no invasive pattern could be observed [33]. This further supports the importance of using primary GSCs to mimic the behaviour of the tumour for clinical relevance.

### 4.1. Site of Cell Injections

When it comes to the study of brain tumours, such as glioblastoma, the use of orthotopic injections of either cell lines or patient-derived cells into the brains of zebrafish is becoming standard [14]. However, injecting cells into the brain, especially the small brain of the zebrafish embryo, entails an increased risk of brain damage, which demands a high level of technical skills during the procedure. The small brain is also a limitation for the volume and number of cells that can be injected in comparison to injections at other sites of the larvae. Despite the above-mentioned physiological and technical issues, the potential of studying cells in their right milieu, including cell-to-cell interactions and influences from the microenvironment, make orthotopic injections superior to other injection sites. Classical growth patterns for GBM such as perivascular infiltration, perineuronal tumour cell accumulation and leptomeningeal accumulation are all important histopathological features and can only be studied in the brain, stressing the need for relevant orthotopic injection models [37].

### 4.2. Temperatures for Implanted Fish and Human Cells

In our study, we decided to keep larvae at 34 °C, since keeping larvae at a higher temperature than normal has shown to increase the overall viability among injected GSC. In a study by Cabezas-Sáinz et al., water temperatures of 34 °C and 36 °C were compared. Cells injected into larvae and kept at 36 °C showed better proliferation [38]. However, the challenge when using zebrafish for human xenograft experiments is the difference in environmental temperatures between zebrafish and human cells. The effects of the increased water temperature for the embryos/larvae were evaluated prior to the injection experiment. We found no visible alterations in the developing larvae due to the increased temperature at 34 °C up to 11 dpf. This has also been shown in previous studies by Kimmel et al. describing the different stages of the zebrafish embryo showing a normal development of embryos kept between 25 °C and 33 °C [39]. Xenotransplanted larvae have also been shown to have a high survival rate of 95% and 87.6% when kept at 34 °C and 36 °C, respectively [20].

In contrast to studies indicating no malformations or developmental abnormalities, there are studies that point out the difficulties in housing embryos and larvae at increased temperatures. Pype et al. increased temperatures by 4–5 °C and showed tail malformations that became more prominent at even higher temperatures up to 36.5 °C [40]. The same study also showed cardiac and head malformations at temperatures around 36 °C. An increased developmental rate was also visible in embryos, leading to earlier hatching at increased temperatures by 2–6 °C. It has also been shown that embryos incubated at 36 °C at 48 h post-fertilisation had a higher mortality than the control groups kept at 28 °C and 34 °C. The metamorphosis process that larvae undergo between 12 and 14 dpf where mortality is often elevated is also affected and pushed to an earlier time point due to rapid development at higher temperatures [41]. Taken together, increased temperatures in the developing larvae should not be neglected, and could affect the outcome of experiments carried out under these conditions.

Although holding temperatures for the host animals have been evaluated, studies have reached no consensus on whether acclimatisation of the tumour cells prior to injection affects the result. In our study, the number and volume of cells at 1 dpi were larger in animals with cells held at 37 °C prior to injection, although this difference was absent by 5 dpi, showing approximately the same number of cells from both temperatures. Surviving cells could possibly have acclimatised by this time to the new environment in the fish brain. In cell culture, the lower temperature resulted in a decreased proliferation rate and changed morphology. Previous studies have described more prominent differences; Eden et al. and Lally et al. found it necessary to decrease the temperature of cell cultures to meet the fish holding temperature not to cause tumour cell death in situ [16,42]. Others, such as Welker et al. and Ahlmstedt et al., have not reached the same conclusion, but instead injected cells into the larvae brain without acclimatisation, still with a good take-rate of tumour cells [26,27].

### 4.3. GSC Labelling Methods

In our study, we evaluated two different labelling methods. Since similar studies have been performed using either labelling technique, i.e., fluorescent protein expression [16,17,26,27,43] and synthetic membrane dyes [43,44,45,46], our goal was to reach consensus in the labelling method. Labelling using membrane dyes, such as DiD, prior to injection avoids time-consuming transfections with genes coding for fluorescent proteins. When working with primary cells, a transfection might not even be a possible option. In addition, to obtain better transfection efficiency, selection medium or single cell cloning can be used, though the resulting culture will present a skewed selection of the cell population used in the experiment. Nevertheless, transfections with fluorescent proteins are well known in the field and the side effects are overlooked. Since membrane dyes fade upon cell proliferation and time, shown by Vargas-Patron et al. in a study using CellTrace [44], the need for a stable marker makes continuous transcription of a fluorescent dye a tempting alternative.

Loss of labelled cells was observed in our injected larvae as well, although GFP-expressing cells decreased faster, presumably because the cells stopped producing the GFP when adjusting to the new environment. This has also been shown in a study by Vargas-Patron et al., where the GFP signal reached a peak around 72 h but then started to decline [44,47]. In our study, this effect was not seen among membrane-labelled cells where DiD-labelling lasted for at least a week. We did observe a difference in the number and volume of cells between these two methods; we therefore chose to label cells with a membrane dye rather than a fluorescent protein.

### 4.4. Length of Observation

Previous xenotransplantation studies of glioma cells have varied in age of the fish used for injection, from blastula stage to juvenile fish, and the durations of observing the tumour cells have varied in length. We focused our analyses on differences between 1 and 5 dpi, and also reported findings from 8 to 9 dpi. Many studies using zebrafish as a xenograft model work by an experimental setup window of 2–4 dpi. This has been shown to work well for many groups injecting human glioma cells into the larvae [43,48,49,50]. Although this experimental setup is easy and fast, longer duration of monitoring glioma cells in the larvae is needed for toxicity testing and drug screens of slow-acting drugs, such as epigenetic drugs. However, one should also take into account that paediatric and adult GBM have different molecular features that could possibly affect the outcome of growth. Additionally, the difference between cell lines and primary cell cultures should be emphasised.

When studying the growth pattern and drug response of the tumour cells, duration and acclimatisation of the tumour cells are important. At the short time span of 5 dpi in our experiment, it was a challenge to detect cell-specific differences between our patient GSC lines, compared to the previous mouse study [10]. In the zebrafish, such observations are hard to make since the timeline for the progression is too short.

Moreover, the importance of cell proliferation and stable cells over time in the larvae brain is important, since most clinically used treatments target proliferating cells. The observed drifting from round to star-shaped cells that can be seen at 5 dpi in our study could potentially indicate a differentiation and therefore also cell cycle arrest. This topic needs to be further investigated to develop a better understanding of this paediatric glioblastoma model.

### 4.5. The Role of Immune System

Cell death due to an immune reaction could be one of the reasons for the loss of cells with time seen in our experiment. Although a mature immune system is not fully developed in larvae up to 14 dpf, a first line of defence with macrophages and dendritic cells is already present after a few days [12]. Because of the lack of a full immune system, most studies have been carried out before this critical time point. Nevertheless, since the larvae are not immune-suppressed, the reason for the loss of tumour cells might be due to the innate immune system of the animal. A study by Lally et al. showed that larvae can be immune-suppressed using irradiation prior to experiments run for up to 5 dpf without any effect on the development, although this procedure would add further steps to the protocol [41,42]. Prior to our study, in an attempt to rule out the possibility of cell loss due to an immune response, we tested if dexamethasone, a chemical previously used to suppress the immune system in adult zebrafish by Eden et al. [16], could also be used for larvae. Unfortunately, this approach had to be rejected since the compound was toxic to the larvae and caused animal death. Since Welker et al. have shown tumour survival up to 25 dpf without rejection [26], we concluded that immune suppression of the animals was not required. However, a recent publication by Yan et al. developed an immunocompromised adult zebrafish strain (*prkdc^−/−^*, *il2rgα^−/−^*) lacking B, T and natural killer cells and housed at 37 °C. In this model, temperature differences are not an issue, which makes the model a suitable PDX model for human tumour cells [51].

### 4.6. Pharmacological Treatment Effects

In our drug experiment in vitro, treatment of GSCs with TMZ and the epigenetic drug VPA showed increased efficacy using the combination of drugs based on cell viability. This is in accordance with previous studies using commercial glioma cell lines [29]. Treatment with TMZ in combination with decitabine showed a decrease in proliferation and viability, but a synergetic effect was only seen in one of the cell lines. Treatment with decitabine alone resulted in differentiation, as previously shown in adult glioma cell lines [52], which could explain the non-synergistic combination effect since cells differentiated and stopped to proliferate, which would decrease the effect of the drug since it depends on cells cycling to incorporate into the DNA, rather than entering apoptosis. Hence, there was a significant decrease in the number of cells in our combined treatment, which confirmed results seen in previous studies [53]. The differed response among the cultured cell lines emphasises the importance of using patient-specific primary cell cultures to give guidance on treatments in further experiments.

To perform a proof-of-concept experiment of our model using primary GSCs, we treated injected larvae with TMZ, VPA and decitabine alone and in combination. When evaluating the survival of injected GSCs over time, we could observe a decrease in cell number across all drugs and an even greater effect on the cell volumes. Treatment with decitabine resulted in a smaller effect on the total cell number. This might be explained by the short time span and the actual effect of the drug being dependent on incorporation into the DNA [54]. If cells divide slower or stop dividing when entering the zebrafish brain, the primary effect of the drug is lost. VPA, on the other hand, is a deacetylase inhibitor that works in a different way. In a study by Catalano et al., treatment with VPA induced cell cycle arrest and apoptosis in thyroid cancer cells [54,55]. Morphological changes in GSCs treated with VPA in vitro shown by Riva et al. could also partly be observed in our study in vivo [56]. We could also see a greater treatment effect using VPA in our experiments. Overall, one point to acknowledge is the impact of water temperature. Cabezas-Sáinz et al. showed that treatment using the anti-cancer drug 5-fluorouracil had better effects at higher water temperatures of 36 °C compared to 34 °C [38]. This issue should include all above stated studies where water temperatures were between 32 °C and 34 °C.

Zebrafish larvae have been used in treatment studies using TMZ for brain tumours in the past. Geiger et al. showed that larvae injected with human GBM in the yolk sac responded to pre-treatment of TMZ in combination with radiation. They also showed that TMZ alone had no effect on the developing larvae [17]. Moreover, Welker et al. showed responsiveness to chemotherapy such as TMZ, where more than 70% survival up to 25 dpi compared to untreated was seen, with no animal surviving more than 12 dpi [26]. In a more recent paper, Welker et al. showed changed heterogeneity of GBM cells injected orthotopically into zebrafish larvae treated with TMZ. They measured the amount of proliferating, differentiated and stem cell-like cells after 5 and up to 20 days after treatment and concluded that proliferation is halted immediately after treatment with TMZ; the number of vimentin-positive cells was also decreased, though there were no effects on SOX2- and GFAP-positive cells. Interestingly, SOX2-positive cells increased a few days after treatment, indicating a larger population of stem-like cells [57]. Castello et al. also demonstrated that TMZ resulted in significant reduction in tumour cells, and in combination with the anticancer drug Onalespib, found synergistic effects with even better treatment results [58]. Other treatment strategies using other drugs such as VPA, which has been effective in vitro as well as in clinical studies, have also worked effectively as anticancer and antiangiogenic drugs in zebrafish larvae [35]. In our study, we observed that cell volume is affected more than the cell number. This result of decreased cell volume could be due to apoptotic volume decrease (AVD), a cell cycle hallmark indicating cell death. Treatment with drugs affecting the membrane channels of the cells, such as VPA and TMZ, affect the morphology, resulting in decreased cell density and cell shrinkage [55,59].

The potential of using orthotopically transplanted GSCs in larvae as a model for future drug screening of paediatric tumours is still to be fully evaluated. The evaluation of drug responses should therefore ideally be validated using a larger number of animals. However, other studies have also shown that drug response of injected glioma cell lines can be assessed and monitored using zebrafish larvae [29], supporting the future utility of our model.

## 5. Conclusions

To summarise, we have developed a model to study patient-derived paediatric GSCs in vivo using zebrafish larvae. Injected cells established in the brain and could be monitored over 8 dpi. This model could be used for evaluating treatment response early after the diagnosis of the patient and could therefore be of clinical relevance in guiding further treatments for glioblastoma in children.

## Data Availability

The data presented in this study are available on request from the corresponding author.

## Supplementary Materials

The following supporting information can be downloaded at: https://www.mdpi.com/article/10.3390/brainsci12050625/s1, Figure S1: Morphological features of cells grown at 34 °C before xenotransplantation into the zebrafish brain; Figure S2: Proliferation and differentiation of stem cell lines in culture after treatment with drugs; Figure S3: Morphological features of injected GSC lines GU-pBT-7 (A) and GU-pBT-19 (B) in the larval brain after treatment with combinations of TMZ, VPA and decitabine.
