# Supplementary material for: Orthotopic Transplantation of Human Paediatric High-Grade Glioma in Zebrafish Larvae
Source: Brain Sci. 2022 May 10;12(5):625. doi: 10.3390/brainsci12050625 (PMC9139401; doi:10.3390/brainsci12050625)
Supplement: Supplementary file 1 [file brainsci-12-00625-s001.zip › brainsci-1655362-supplementary.pdf]

Orthotopic transplantation of human paediatric high-grade glioma in zebrafish larvae

Susanna Larsson, Petronella Kettunen and Helena Carén

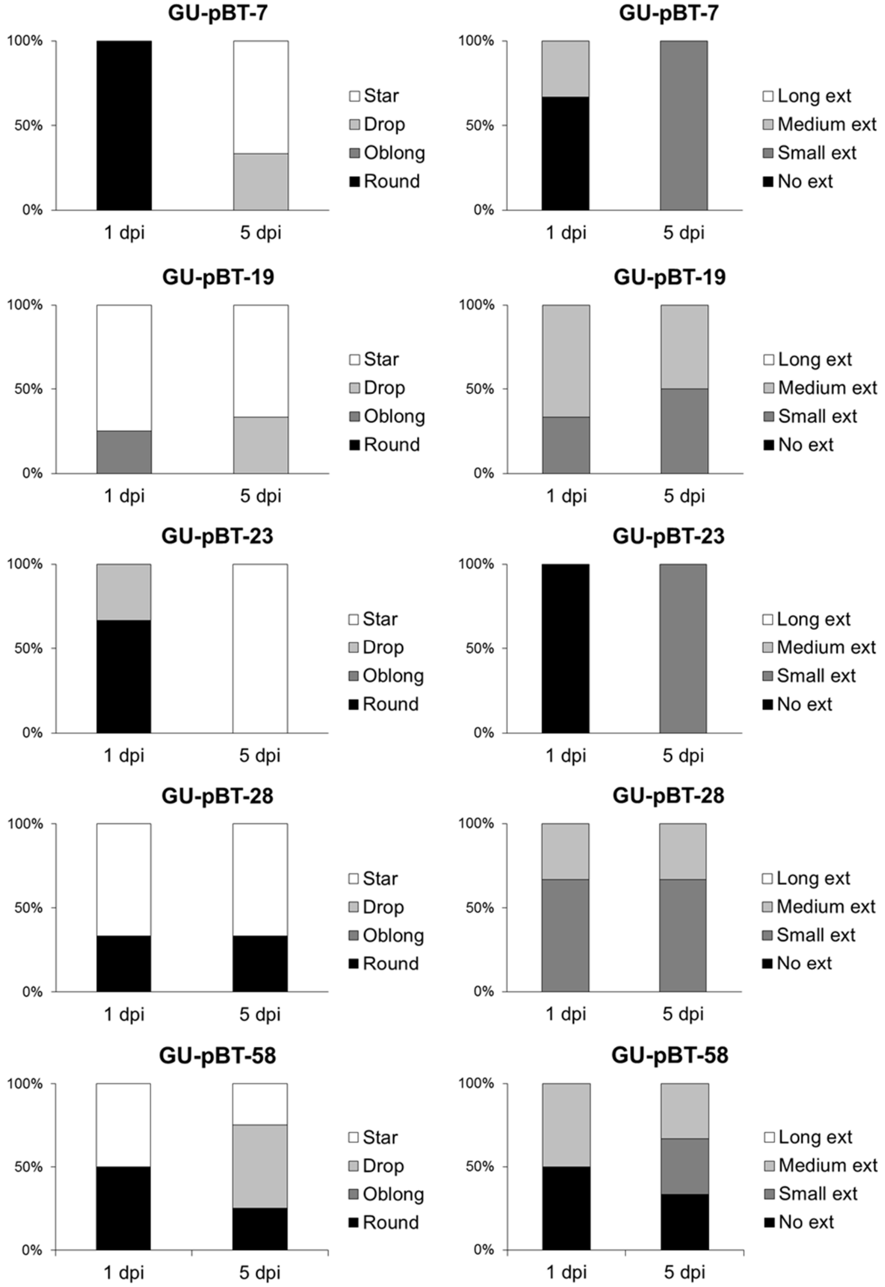

Supplementary Figure S1. Morphological features of cells grown at 34 ˚C before xenotransplantation into the zebrafish brain. The effect of lower temperature could be observed in the morphology of cells *in vivo*. The high presence of round cells with no or short extensions (ext) could be a sign of cells in the state of cell death. Star-shaped cells on the other hand were likely reaching out, starting to invade the surrounding. Interestingly, we did not observer any long extensions of cells grown at 34 ˚C after they had been xenotransplanted. Dpi: days post injection.

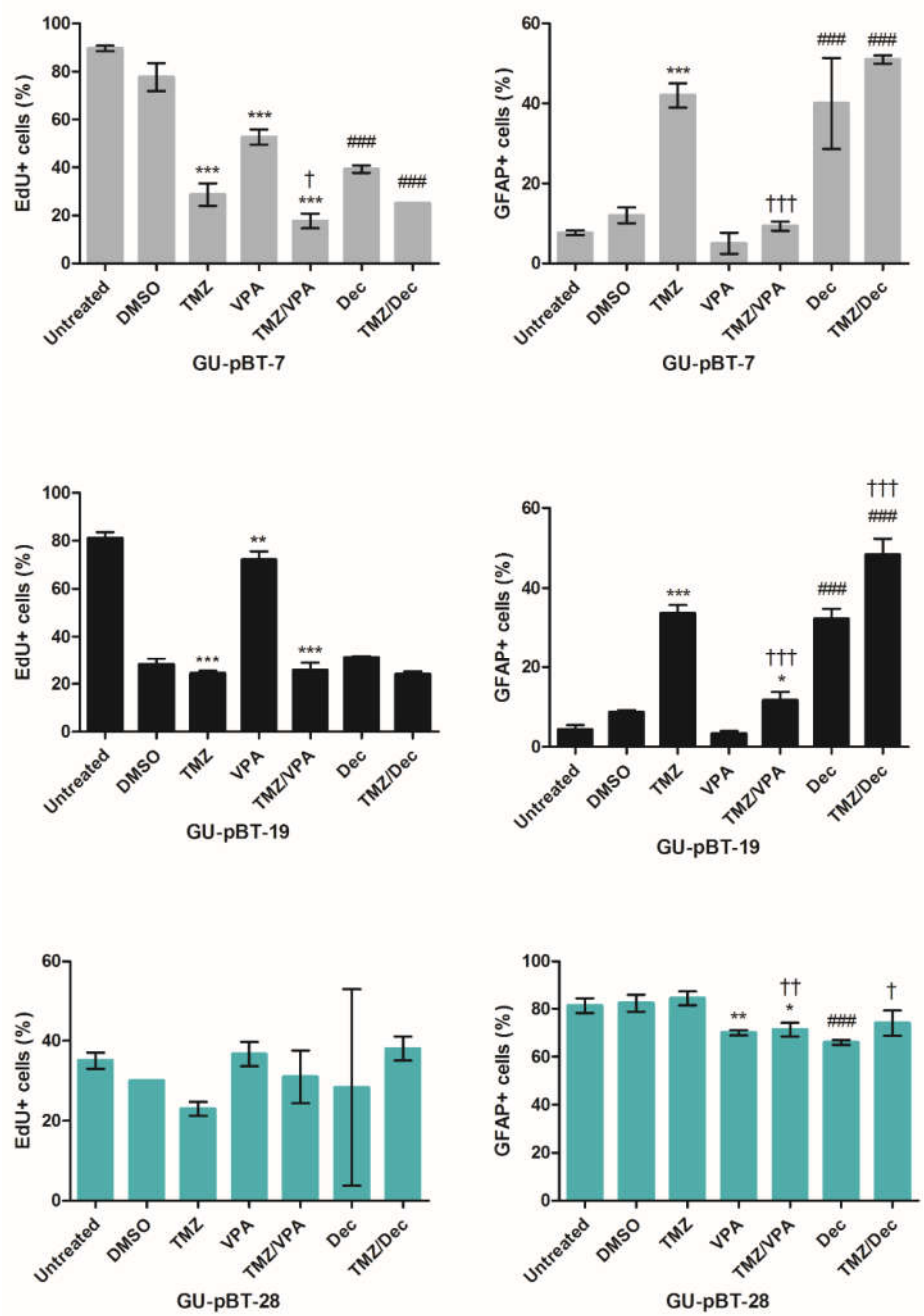

Supplementary Figure S2. Proliferation and differentiation of stem cell lines in culture after treatment with drugs. Proliferation was assessed as EdU+ cells and differentiation as GFAP+ cells. TMZ: temozolomide; VPA: valproic acid; Dec: decitabine. Data is presented as means and standard errors. * *p*-value < 0.05, ** *p*-value < 0.01 and *** *p*-value < 0.001 correspond to significant differences between untreated vs. TMZ, VPA and TMZ/VPA, respectively. ### *p*-value < 0.001 corresponds to comparisons of DMSO control vs. Dec and TMZ/Dec. † *p*-value < 0.05, †† *p*-value < 0.01 and ††† *p*-value < 0.001 correspond to comparisons of TMZ vs. TMZ/VPA and TMZ/Dec, respective

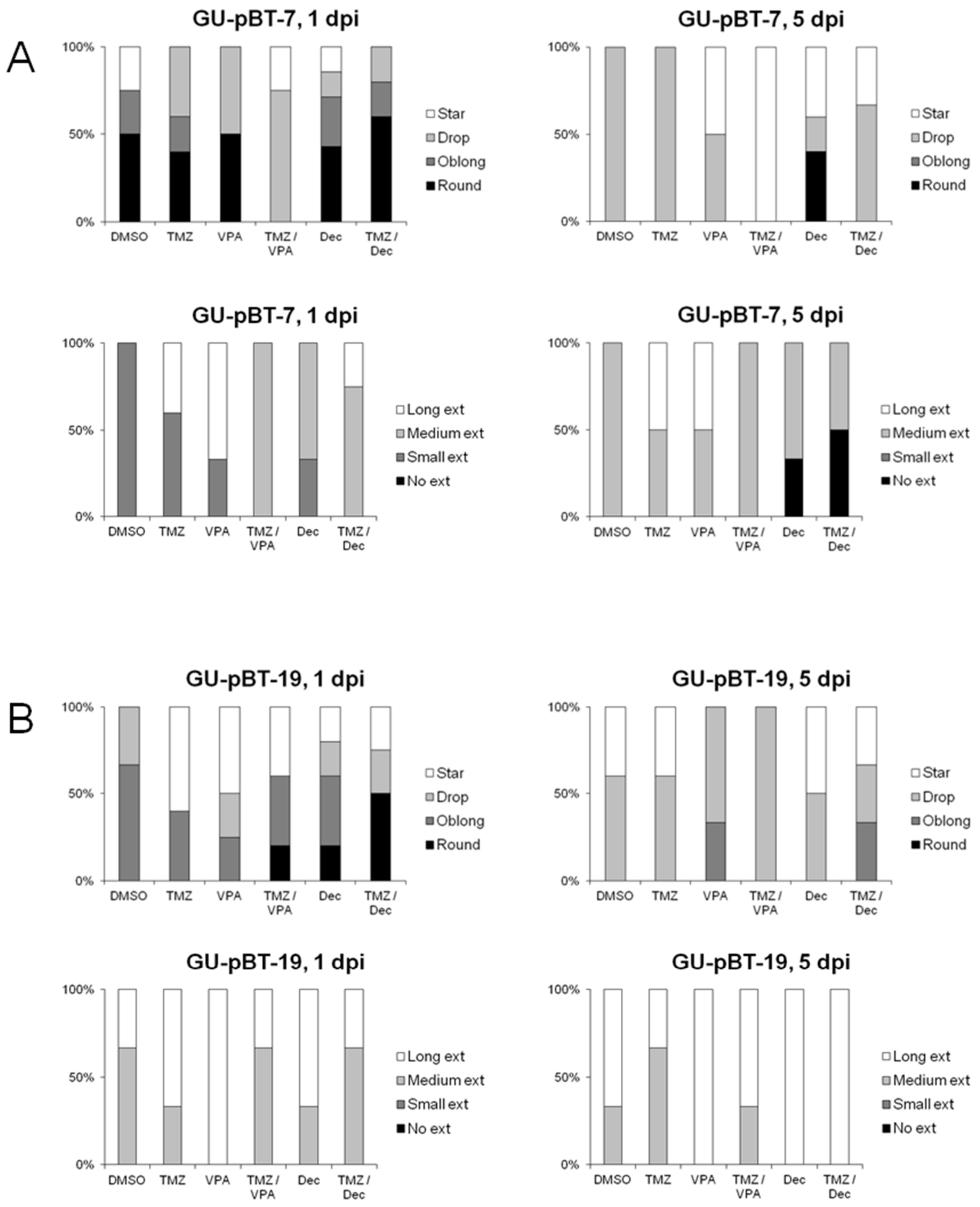

Supplementary Figure S3. Morphological features of injected GSC clines GU-pBT-7 (A) and GU-pBT-19 (B) in the larval brain after treatment with combinations of TMZ, VPA and Dec. Cell morphology and neurite length was observed at 1 dpi and 5 dpi. Graphs depict the percentage of fish displaying injected cells with the specific morphology. Dpi: days post injection; ext: extensions; TMZ: temozolomide; VPA: valproic acid; Dec: decitabine.
